# Identifying the Most Critical Predictors of Workplace Violence Experienced by Junior Nurses: An Interpretable Machine Learning Perspective

**DOI:** 10.1155/jonm/5578698

**Published:** 2025-04-02

**Authors:** Lanjun Luo, Yuze Wu, Siyuan Li, Fengling Li, Xueyan Wang, Xuemei Wei

**Affiliations:** School of Management, Affiliated Hospital of North Sichuan Medical College, North Sichuan Medical College, Nanchong, China

**Keywords:** critical predictors identifying, interpretable machine learning, junior nurses, risk prediction, workplace violence

## Abstract

**Background:** Workplace violence, defined as any disruptive behavior or threat to employees, seriously threatens junior nurses. Compared with senior nurses, junior nurses are more vulnerable to workplace violence due to inexperience, low professional recognition, and limited mental resilience. However, there is an absence of research discussing the workplace violence risk of junior nurses, in particular, the lack of analysis of critical factors within the multiple influences and the lack of targeted risk prediction models.

**Objective:** Considering the multiple influencing factors faced by junior nurses, this study aims to predict the risk of workplace violence using interpretable machine learning models and identify the critical influencing factors and their nonlinear effects.

**Design:** An observational, cross-sectional study design.

**Participants:** A total of 5663 junior registered nurses in 90 tertiary hospitals in Sichuan Province, China.

**Methods:** Data are all obtained through a questionnaire survey. An interpretable machine learning framework, including the Light Gradient Boosting Machine (LightGBM) model and two post hoc interpretable methods, Accumulate Local Effect and SHapely Additive exPlanations (SHAP), are conjoined.

**Results:** The LightGBM model is more accurate than other machine learning methods, achieving an area under the receiver operating characteristic curve of 0.761 and a Brier score of 0.198 on the workplace violence prediction task. Among the dozens of potential influences input into the predictive model, seeing medical complaints, psychological demands, professional identity, etc., are the most critical predictors of workplace violence.

**Conclusions:** The proposed LightGBM-SHAP-ALE approach dynamically and effectively identifies junior nurses at high risk of workplace violence, providing a foundation for timely detection and intervention.

## 1. Introduction

Workplace violence, defined by the International Labor Organization as threats, injuries, or unreasonable acts of physical, psychological, or verbal abuse occurring during work [1], poses a critical challenge to nursing professionals globally [2]. As frontline healthcare providers, nurses experience disproportionately high workplace violence exposure, with studies indicating over 75% encounter such incidents [3]. Workplace violence can severely impact nurses' physical and mental health, hinder their professional growth, and consequently lower the quality of healthcare and patient safety. Hence, implementing early prediction systems and robust preventive strategies to reduce workplace violence incidents is crucial for safeguarding nurses' welfare and maintaining a stable healthcare system.

Based on such considerations, contemporary mainstream healthcare workplace violence research mainly focuses on two topics: risk prediction and cause analysis. Risk prediction focuses on assessing workplace violence in healthcare places' frequency, probability, etc. [4–6]. Cause analysis research focuses on what leads to workplace violence and the most important predictors that reveal the risk of workplace violence [7–9]. However, current research predominantly examines workplace violence among indistinctive nurse groups, neglecting differences in seniority levels.

Workplace violence against junior nurses is unique. First, the relative risk is higher, with healthcare workers having less than 3 years of experience being about 30% more likely to face workplace violence than other groups [10]. Second, the low income and lack of professional identity make it challenging for junior nurses to provide satisfactory healthcare services [11], further contributing to workplace tensions. Third, junior nurses are particularly vulnerable to being targeted by senior colleagues for professional bullying [12]. Fourth, junior nurses also lack the experience and ability to deal with workplace violence [13, 14]. Moreover, the consequences of workplace violence for junior nurses are more severe and can easily lead to psychological trauma and separation from the workforce [15].

Beyond the insufficient focus on junior nurses in research subjects, there are also several methodological limitations in the related studies. Many studies analyzing the causes of workplace violence have employed parametric models such as logistic regression (LR) and structural equation modeling [16]. However, these approaches typically rely on linear assumptions, making capturing complex nonlinear relationships and variable interactions tough, thus constraining the understanding of individual heterogeneity [17, 18]. Furthermore, these methods are utilized based on hypothesis testing, which requires domain-specific knowledge to specify mediating, moderating, and other relationships in particular contexts, and lacks hypothesis-free causes and mechanism discovering [19].

Therefore, it is crucial to tackle the ensuing questions to bridge the research gap: Why does workplace violence occur among junior nurses? How to accurately predict workplace violence risk? What are the most critical predictors? Due to the influence of various potential factors and the inherent suddenness and randomness of workplace violence among junior nurses, accurately predicting workplace violence risk and identifying key influencing factors from high-dimensional input variables become critical tasks.

Interpretable machine learning can help tackle this challenge. The primary objective of interpretable machine learning is to enhance the transparency and comprehensibility of predictive models [20]. It enables users and decision-makers to comprehend the decision-making logic of these models, thereby allowing intervention in high-risk outcomes. Interpretable machine learning has gained traction in healthcare research, providing a crucial alternative to conventional analytical tools like Nomogram [21, 22].

Against this backdrop, this study aims to predict the risk of workplace violence among junior nurses and identify critical influencing factors and their nonlinear effects using an interpretable framework. Drawing on classical socioecological theory [23], this study gathered 76 predictors related to workplace violence exposure history from 5663 nurses across 90 tertiary hospitals in Sichuan Province, China. Machine learning methods, including Light Gradient Boosting Machine (LightGBM) [24] and eXtreme Gradient Boosting (XGB) [25], were used for prediction. Specifically, the study adopted the SHAP [26] to ascertain the pivotal predictive factors at the global and individual sample levels. In addition, the accumulated local effects (ALEs) [27] method was implemented to simulate controlled variable manipulation, thereby dissecting the mechanisms and patterns through which critical features exert their influence on the risk of workplace violence.

The contribution of this study can be summarized as three-fold as follows:

a. A LightGBM-SHAP-ALE-based interpretable machine learning framework is proposed for junior nurses' workplace violence risk analysis.
b. Nurses' subjective feelings and work situations are found to have the most significant impact on workplace violence compared with demographic factors.
c. The nonlinear relationships between influencing factors and workplace violence are found using the ALE method, giving support to workplace violence risk management strategies.

## 2. Literature Review

Research on workplace violence has long focused on critical explanatory predictors and their effects. Extensive reviews focus mainly on patient, staff, environmental, and sociocultural factors [28, 29]. Healthcare workplace violence-related studies have categorized influencing factors similarly [30–32]. Typically, Jonsdottir et al. [33] used Binomial and Poisson regression to analyze the risk factors for women's exposure to workplace violence, in which eight variables, such as income, working hours, and shifts, have significant effects. Sun et al. [34] employed LR to identify risk factors for healthcare workers' exposure to workplace violence. The analysis revealed that eight variables significantly impact this exposure, including years of experience, night shift duration, self-reported work environment, and social status. Qi et al. [35] analyzed the risk factors of workplace violence suffered by medical staff during the COVID-19 epidemic by the LR method. Among them, seven variables, such as working years, direct contact with COVID-19 patients, self-discovery of medical errors, depression, and anxiety, were significant. Nøland et al. [36] analyzed medical students and doctors based on two national teams in Norway using repeated measures analysis of variance. Detailed information on primary related literature is shown in Table 1.

However, as previously mentioned, the prevailing methods, including LR and several classical econometric models, although renowned for their computational clarity and favorable efficacy in hypothesis testing, these models have encountered challenges in recent years. Particularly in risk analysis domains such as conflict [17, 19] and crime [43], which are akin to workplace violence research, traditional models typified by LR exhibit inferior predictive accuracy compared with contemporary machine learning models. With its adeptness in capturing nonlinear relationships and handling complexity, machine learning has progressively found application in nursing and workplace violence research.

Thus, in recent years, there has been an increase in the use of machine learning in the field of healthcare workplace violence. Representatively, Havaei et al. [44] used machine learning models such as the random forest (RF) algorithm to predict different types of workplace violence and analyze the most predictive factors. Lee et al. [6] adopted machine learning models such as decision trees, Naïve Bayes, and RF to predict workplace violence in emergency departments using electronic health record data. Hu et al. [45] used machine learning methods such as support vector machines and artificial neural networks to predict violence in psychiatric inpatients. Kirchebner et al. [7] used the support vector machine, decision tree, K-nearest neighbor algorithm, and other machine learning models to examine the causes of violent offending in individuals with schizophrenia spectrum disorders.

Nonetheless, machine learning models are perceived as “black boxes,” requiring supplementary techniques for explainability [46]. With the advancement of machine learning techniques, particularly in complex models such as deep neural networks, a mounting concern arises regarding their less interpretability nature [47, 48]. Complicated machine learning models can acquire intricate and nonlinear relationships between input features and output predictions, posing challenges for human comprehension of the decision-making process behind specific predictions.

Interpretable machine learning strives to clarify complex models, making them more transparent and easier to understand through the development of specialized methods. Typical techniques include the Permutation Feature Importance analysis [49], Local Interpretable Model-agnostic Explanations (LIME) [50], and SHapley Additive exPlanations (SHAP) [26], which have been adopted in many risk analysis-related works [51, 52]. Thus, many recent nursing studies have applied these technologies due to the advantages of interpretable machine learning. For example, Lei et al. [53] used the XGBoost as the primary prediction model to analyze the delirium risk for critically ill children, while the logistic-based nomogram visualization is chosen as a proximate explanation. Zhou et al. [54] adopted SHAP to enhance the model explainability.

Nevertheless, related works mainly focus on global-level explanations, which means the overall pattern and feature-based attributes on the entire dataset [20]. Global interpretability complicates the analysis of risk formation mechanisms and outcomes across samples. It fails to address questions like, “Why is this nurse at a higher risk for workplace violence?” or “Why do two nurses in the same department have significantly different risk predictions?” Thus, there is a need to utilize instance-level interpretability.

Furthermore, current explanations mainly concern feature importance, yet there is a lack of reporting on how different features affect the output, where linear or nonlinear effects need to be reported. This problem needs to be addressed similarly to control variables, and in particular, the net effect of the variable of interest on risk needs to be isolated, controlling for other covariates. Therefore, the ALEs [27] interpretation needs to be employed, which can provide nonlinear relationships that are difficult to find in parametric models such as the traditional LR, and it can compute the net effect of the specified features on the output.

In summary, based on the literature review, this study aims to identify the most critical predictors of workplace violence experienced by junior nurses. Interpretable machine learning techniques are used to gain more care management insights by accurately predicting risk while analyzing the net impact effect using SHAP instance-level interpretation and the ALE method.

## 3. Materials and Methods

### 3.1. Study Design and Participants

This study used a multicenter cross-sectional research design and a multistage proportional random sampling method to randomly select junior registered nurses from tertiary-level hospitals in each city of Sichuan Province from September 2021 to October 2022, and 5663 junior registered nurses were finally selected from 90 tertiary-level hospitals. The operational definition of junior nurses in this study is: nurses with ≤ 5 years of working time and no working experience (except for clinical internships) before joining the hospitals (relevant references are attached). Inclusion criteria were as follows: ① nurses who joined in 2016, i.e., working time ≤ 5 years and no working experience before joining; ② nurses with practicing certificates; ③ voluntary participation in the study and signing of the online informed consent form; and ④ age ≥ 18 years old. Exclusion criteria were as follows: junior nurses who had a major stressful event such as the death of an immediate family member, suffered a major surgery, suffered from a major disease within the last 6 months, or were currently receiving psychotropic drugs and psychotherapy.

### 3.2. Variables

#### 3.2.1. Measurements of Workplace Violence Experienced by Junior Nurses

The prediction target for this study was whether or not the junior nurses had experienced workplace violence, with the outcome variable named *Violence.* In the study design, *Violence* was coded as 1 whenever violence was personally experienced or witnessed and 0 when neither occurred.

In detail, the Workplace Violence Scale (WVS), developed by Schat and Kelloway [55] and Chinese version localized by Wang [56], was used to investigate workplace violence experienced by the study participants in the past year, including information on physical assault, emotional abuse, threats of intimidation, verbal harassment, and sexual harassment, and the Cronbach's alpha of the scale was 0.992. Each item is rated on a 4-point scale reflecting the frequency with which the participant has experienced workplace violence in the past year (1 = never, 2 = once, 3 = two or three times, and 4 = four or more times), with a total score of greater than five indicating exposure to workplace violence. This study also utilized the scale to investigate the participants' witnessing of others experiencing workplace violence in the past year; similarly, a total score greater than 5 indicated witnessing workplace violence.

#### 3.2.2. Predictors

Based on the socioecological theoretical model, factors affecting workplace violence can be classified into intraindividual levels, such as demographics, nurse-related and patient-related factors; interpersonal levels, such as the nurse–patient relationship; organizational levels, such as organizational support; and social-policy levels, such as governmental or hospital policies and social culture. The five levels are interconnected and contribute to the occurrence of workplace violence. To better analyze the different aspects of factors influencing workplace violence, following the fundamental study of Caruso et al. [30], which similarly divided the workplace violence risk factors into five subsets, including the workplace and policy aspect, the patient aspect, the physician aspect, the doctor–patient relationship aspect, and the sociocultural aspect, this study also obtains prediction input variables from such discrimination. The obtained variables are listed in Table 2. The first column lists the names of each variable, and the categorical variables further record the different categories' meanings after indentation. The second column shows the descriptive statistics for each variable, recorded as counts (percentages) for categorical variables and means (standard deviations) for continuous variables.

**Figure 1:**
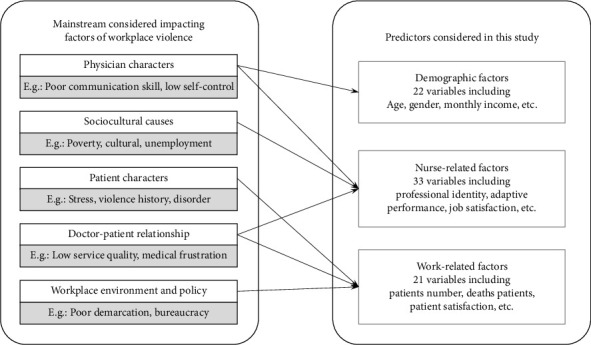
The predictor filtering framework in this study.

**Figure 2:**
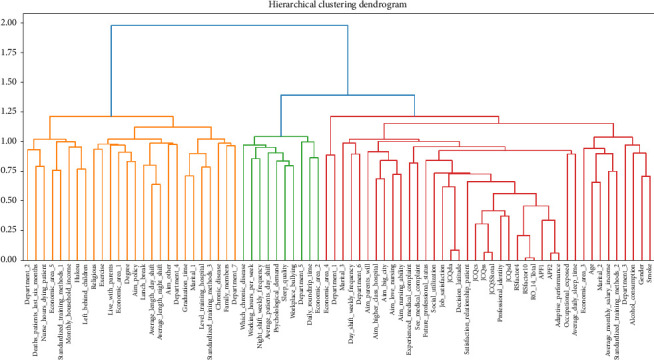
Hierarchical cluster analysis of predictors in this study.

Considering the patient privacy reasons and the difficulty of obtaining relevant data, this study focused on three main aspects of screening influencing factors of workplace violence among junior nurses. As shown in Figure 1, the first category is demographic factors, mainly age, gender, etc., of each junior nurse, which are unrelated to their work situation and subjective feelings. The second category is nurse-related factors, emphasizing subjective feelings such as professional identity and job satisfaction. The third category is work-related factors, which include variables that measure the quality of nursing services and the nurse–patient relationship, such as the number of patients, the number of patient deaths, and patient satisfaction. The descriptive statistics, univariate Chi-square, and *t*-test results can be seen in Table 2; statistically significant variables are bolded. The specific meanings and measurements of all variables are shown in detail in the Supporting Information 1. The four features, including *Economic_area*, *Marital_status*, *Standardized_training_methods*, and *Department*, were one-hot coded with dummy variables. In addition, this study used a feature hierarchical clustering approach to categorize all the used predictors, as shown in Figure 2. The predictors were also categorized into three major categories, which is consistent with the theoretical analysis.

### 3.3. Machine Learning Models and Post Hoc Explainations

**Figure 3:**
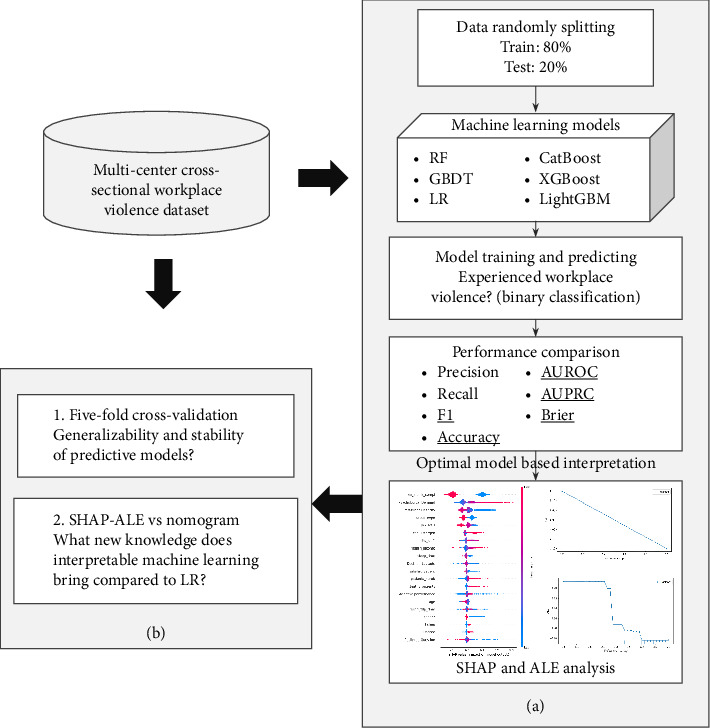
Overall predictive analysis framework. (a) Baseline predictive analysis and (b) robustness analysis.

The framework for analyzing the overall prediction of workplace violence among junior nurses in this study is shown in Figure 3. First, the multicenter cross-sectional study data were randomly divided, of which 80% was used for the training set and 20% for the test set. Next, multiple machine learning models were used for training and prediction on a binary categorization task of whether or not experienced workplace violence. The generalization ability and stability of the model are assessed by comparing the performance metrics of the model, such as accuracy and F1 score, and then the optimal model is used for interpretable analysis. In addition, the study conducted a robustness test to check the reliability of the predictive model through five-fold cross-validation and also compared the interpretable results with LR-based nomogram plots to analyze the potential novel findings.

In this study, six algorithms are mainly employed for risk prediction, which are used to screen the most efficient models for interpretable analysis. The algorithms can be divided into three categories: tree-based, neural network-based, and traditional LR. First, facing challenges such as possible feature interactions or covariances with a large number of inputs, tree-based models with excellent robustness on tabular tasks become the most appropriate choice. Therefore, the five well-known models: LightGBM, XGBoost [25], RF [49], Gradient Boosting Decision Tree (GBDT) [57], and CatBoost [58], are chosen to represent the tree-based models. To demonstrate the task adaptability of machine learning models, LR was also used in this study for comparison.

Moreover, this study mainly adopted SHAP and ALE post hoc interpretation methods to explain the model decision process. SHAP focuses on explaining the importance of each feature in contributing to the final prediction at the instance level, and the aggregated effects also yield a global interpretation of the entire dataset. The computational procedure is not derived here because SHAP has been applied in several nursing studies [51, 54, 59]. Unlike SHAP, which mainly provides feature importance and additivity effect, ALE focuses on explaining how features affect the model prediction on average when fixing other inputs. Through selecting an interesting input feature, ALE divides the samples into different bins and makes forecasting using the prediction model and specific samples in each bin.

### 3.4. Prediction Measurements

To comprehensively evaluate the performance of different machine learning models, this study adopted several general classification measurements, including the recall, precision, F1-score, area under the receiver operating characteristic curve (AUROC), area under the precision recall curve (AUPRC), and the Brier score. The meaning and calculation of these measurements are as follows in Table 3: TN, FP, FN, and TP are the four basic statistics evaluating the relationship between model predictions and actual values in binary classification tasks. Based on this, F1, recall, and precision can be calculated as shown in equations (1)–(3). Furthermore, AUROC, AUPRC, and Brier scores are the most used measurements in imbalance classification tasks.

Based on the four fundamental quantities, other measurements can be calculated as follows:

(1) $$\begin{array}{c} \text{Precision}=\frac{\text{TP}}{\text{TP}+\text{FP}}, \end{array}$$

(2) $$\begin{array}{c} \text{Recall}=\frac{\text{TP}}{\text{TP}+\text{FN}}, \end{array}$$

(3) $$\begin{array}{c} F1=2*\frac{\text{Precision}*\text{Recall}}{\text{Precision}+\text{Recall}}. \end{array}$$

### 3.5. Ethical Approval and Informed Consent

This study was approved by the Ethics Committee of the Affiliated Hospital of North Sichuan Medical College (2021ER006-1). All participants participated in the survey voluntarily and signed a web-based informed consent form. Participants' privacy and personal information are protected throughout the study. To protect participant confidentiality, all personal data collected in the study will be used for research purposes only and stored on a secure server with access restricted to research team members. At the end of the study, all personally identifiable information will be destroyed, ensuring that the participants' privacy is protected over time. Furthermore, each research team member has executed a confidentiality agreement, pledging to preserve the confidentiality of sensitive data acquired throughout the study's tenure.

## 4. Results

### 4.1. Model Prediction Performances

**Figure 4:**
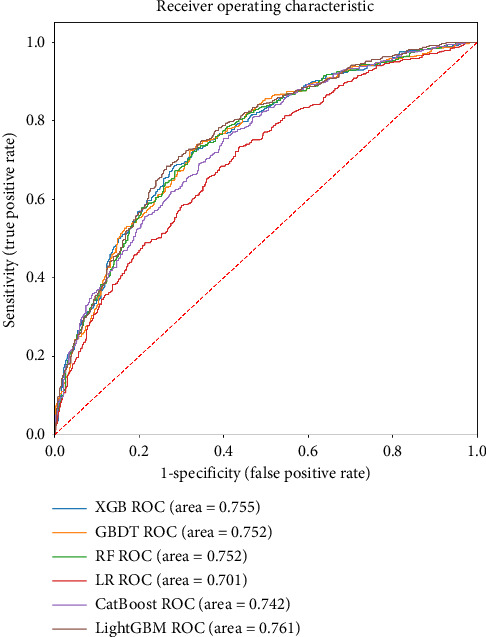
ROC curves of all utilized machine learning prediction models.

In the supervised prediction task, 80% of the samples are randomly selected to build the training dataset, while the remaining 20% are covered in the test dataset for model validation. The model comparison results are shown in Table 4, and the receiver operating characteristic (ROC) curves of different models are plotted in Figure 4. Except for the Brier score, higher scores on the other five assessment metrics indicate better model performance. Considering that the proportion of positive samples (*Violence* = 1) in this study is only 40.8% and that samples in which did not experience workplace violence (*Violence* = 0) constitute the vast majority, this is a typical unbalanced classification problem. In addition to the overall accuracy, more attention must be paid to the four indicators more sensitive to this challenge: F1, AUROC, AUPRC, and Brier. As shown in Table 4, the most important measurements are italicized for ease of reading.

In Table 4, models that perform optimally under the most important metrics are bolded. LightGBM achieves the best results on all five essential measurements. The Brier score for LightGBM is 0.198, in the preferred range (< 0.2). For the AUROC and AUPRC, LightGBM achieved 0.761 and 0.692, respectively, both of which are at acceptable levels. In addition, the models that performed relatively better are XGBoost and CatBoost. Furthermore, through the ROC curves in Figure 4, it can be seen that LR, the traditional statistical model for classification, has an AUROC score of 0.701, in which LightGBM improves by 8.6%, while LightGBM improves by 9.4% on AUPRC. Considering the excellent results of LightGBM, it is used as the core model in the following post hoc interpretability section.

### 4.2. SHAP-Based Interpretations

**Figure 5:**
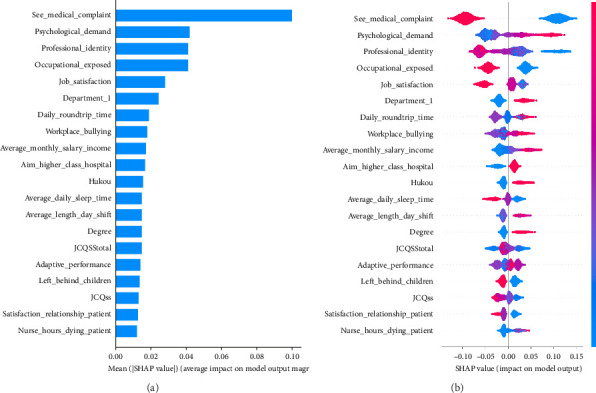
Global-level most important predictors under LightGBM-based SHAP analysis (top 20). (a) Feature importance is shown in the bar plot. (b) Feature importance is shown in the Beeswarm plot.

First, the importance of the input feature is calculated through SHAP and the optimal LightGBM model. The results in Figure 5(a) are presented in the descending order. Due to the large number of input features, only the top 20% of the most important features are shown. It can be seen that features such as *See_medical_complaint*, *Psychological_Demand*, *Professional_identity*, *Occupational_exposed*, and *Job_satisfaction* are the most important. Furthermore, in Figure 5(b), the degree to which each feature affects the model output is shown, where the *x*-axis represents the SHAP value, which can be either positive or negative. Positive values indicate that the feature has an increasing effect on the model's prediction, and negative values indicate a decreasing effect, where each point represents the corresponding feature's value for a data sample, with red representing higher feature values and blue representing lower ones. The further the point away from the centerline, where the SHAP value is 0, the greater the influence of the feature on the prediction.

Observations indicate that a higher *See_medical_complaint* value, specifically 2, yields a negative SHAP value, while a lower value of 1 results in a positive SHAP value. This suggests that the model anticipates an increased likelihood of workplace violence when a nurse encounters a medical complaint. Regarding *Psychological_Demand*, an elevated score corresponds to a higher SHAP value, indicating that the model predicts a heightened risk of workplace violence, thus demonstrating a positive correlation. The variables such as *Professional_identify* and *Job_satisfaction* exhibit inverse relationships with model predictions, with higher values leading to lower SHAP values. This suggests that junior nurses with greater professional identity and job satisfaction face a reduced risk of workplace violence. The Beeswarm plot for the *Occupational_exposed* reveals that nurses experiencing occupational exposure, indicated by a value of 1, are more susceptible to workplace violence, highlighting their vulnerability. *Department_1* indicates nursing rotation through the emergency department, with those having such experience being at an increased risk. In summary, *Professional_identity* and *Job_satisfaction* tend to negatively influence the predicted risk of workplace violence, whereas *Psychological_Demand*, *Daily_roundtrip_time*, *Workplace_Bullying*, *Average_monthly_salary_income*, and other factors positively impact it. *JCQss*, *Satisfaction_relationship_patient*, and *Nurse_hours_dying_patient* have weaker effects on the risk of workplace violence.

**Figure 6:**
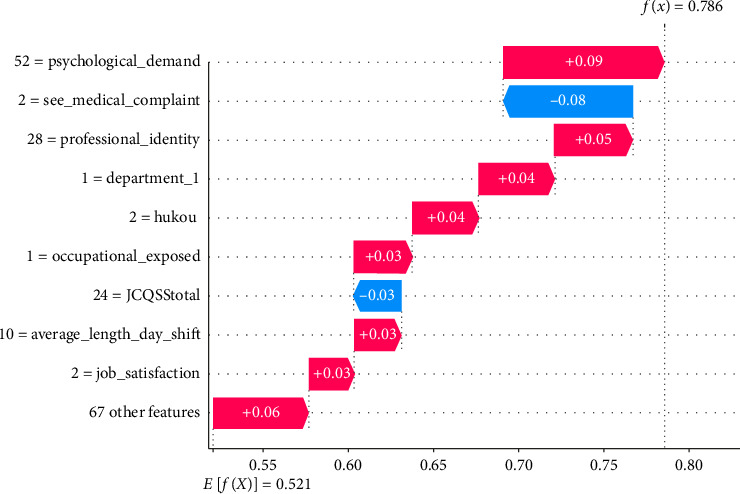
SHAP-based instance-level result of a randomly selected true positive sample.

**Figure 7:**
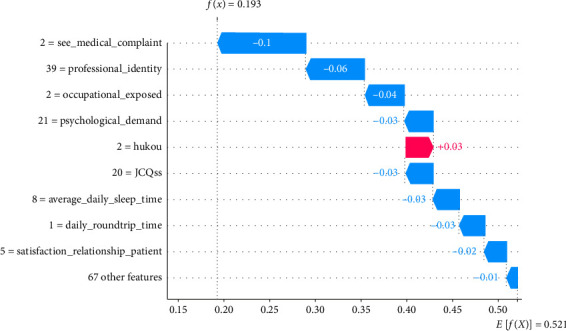
SHAP-based instance-level result of a randomly selected true negative sample.

In the following, individual instance-level analyses were conducted using SHAP, as shown in Figures 6 and 7, which were interpreted for randomly selected TP and TN samples, respectively. At the individual case level, SHAP values indicate the contribution of each feature of that junior nurse's workplace violence risk. The *Y*-axis in the plots represents the values of different features on that sample; for example, *Psychological_Demand* = 52 in Figure 6 means that the corresponding recorded value of this junior nurse is 52. The *X*-axis represents the SHAP value, *E*[*f*(*x*)] represents the expectation of *f*(*x*) for all samples, and *f*(*x*) denotes the predictions. Red features represent features that increase the risk of workplace violence, and blue features represent features that decrease the risk, with larger values and longer arrows representing greater degrees of influence in their prediction.

As shown in Figure 6, only no medical complaints seen (*See_medical_complaint* = 2) and high social support (*JCQSStotal* = 24) reduce the risk of workplace violence. Meanwhile, the remaining eight important features increase the risk, with the model predicting a 79% probability of experiencing workplace violence for this junior nurse, which is much higher than the average expected probability of 0.521. From Figure 7, it can be seen that no medical complaints seen, higher professional identity, no occupational exposure, lower psychological demands, higher leadership support, longer average daily sleep, shorter daily commute, higher satisfaction with patient relationships, and other variables reduced the risk of workplace violence. Thus, the predicted workplace violence probability for this junior nurse sample was 19.3%, lower than the average expectation. Thus, analyzing SHAP values at the individual case level makes it possible to understand how different features affect the model's predictions for each sample. This approach uncovers the inter-individual heterogeneity in risk, particularly highlighting disparities in risk formation and the critical predictors that differentiate among individuals.

### 4.3. ALE-Based Analysis

**Figure 8:**
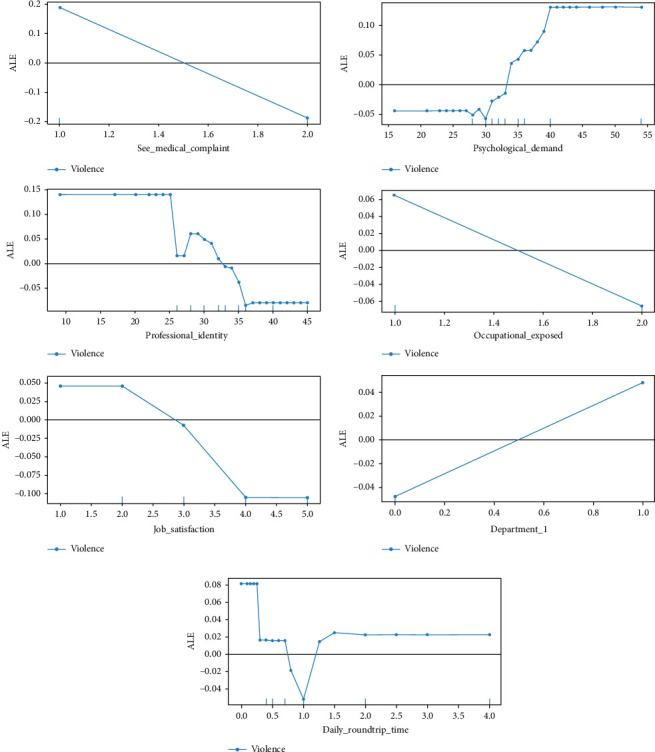
ALE-based analysis of the effects of top-seven important features on prediction.

Based on the SHAP analysis results, this section selected the seven most important features and estimated their impacts on prediction through ALE. In particular, nonbinary variables can be better shown to have a nonlinear association with the predicted outcome. More precise interventions can be provided for nursing management by analyzing overall trends and critical turning points. The results are shown in Figure 8.

The first is the ALE effect of *See_medical_complaint* on the prediction of violence, which can decrease consistently as the value of *See_medical_complaint* increases from 1 to 2. This typical linear effect implies that exposure to medical complaints is significantly associated with the risk of workplace violence among junior primary nurses. Second, it can be observed that the effect of *Psychological_Demand* on workplace violence prediction is nonlinear, with the ALE values going through multiple stages of change as *Psychological_Demand* increases. In the interval of lower *Psychological_Demand*, between 15 and 30, the ALE values stabilize at a low level. Such a phenomenon indicates that at lower levels of psychological demand, the risk of workplace violence is smaller compared to the average. From 30 to 40, the ALE value increases dramatically, significantly enhancing the risk-exacerbating effect. After the *Psychological_Demand* exceeds 45, the increasing trend of ALE values tends to level off but remains high. Overall, there is a nonlinear positive association between psychological demand and workplace violence.

The effect of *Professional_identity* on violence is also nonlinear, with the ALE value dropping abruptly while the *Professional_identity* is approximately 25 and leveling off. This trend suggests that an increase in *Professional_identity* is associated with a decrease in the predicted workplace violence risk. The relationship between *Job_satisfaction* and predicted workplace violence is also similar, with ALE values showing a stabilization–decline–restabilization process. Therefore, particular attention needs to be paid to the turning point around *Professional_identity* = 33 and *Job_satisfaction* = 3, which means that junior nurses are more likely to experience workplace violence if their values are below this range.

In addition, the relationship between *Daily_roundtrip_time* and the risk of workplace violence showed an interesting U-shaped-like structure. *Daily_roundtrip_time* is likely to increase the risk of workplace violence when it is either less than 0.6 h or greater than about 1.2 h, whereas a moderate commute time in between decreases the predicted risk value. It is posited that commute durations that are either excessively short or long may contribute to increased work-related stress, reflecting constraints in housing costs and the selection of workplace locations. This chronic state of elevated stress may render nurses more sensitive and irritable in the workplace, thereby augmenting the risk of workplace violence. Conversely, a moderate commute duration may afford nurses additional opportunities for social interaction, such as engagement with colleagues and facilitating the establishment of social support networks that can mitigate the risk of workplace violence.

### 4.4. Robustness Analysis

#### 4.4.1. Cross-Validation

To deal with the stochastic nature of the machine learning optimization process and to make the results more robust, the widely used k-fold cross-validation approach is used in this study to test all the prediction models. As shown in Table 5, the performance means (standard deviations) for each model are reported based on the results of 5-fold cross-validation, the best performances are bolded. It can be seen that LightGBM is still optimal under the three critical measures of AUROC, AUPRC, and accuracy with a small standard deviation. On the F1 and Brier metrics, the optimal models are XGB and GBDT, respectively, while the Brier value of LightGBM is 0.201, which is only slightly worse than that of GBDT. The abovementioned analysis illustrates the generalization ability and robustness of the LightGBM model in predicting workplace violence risk for junior nurses.

#### 4.4.2. Comparison With Nomogram

**Figure 9:**
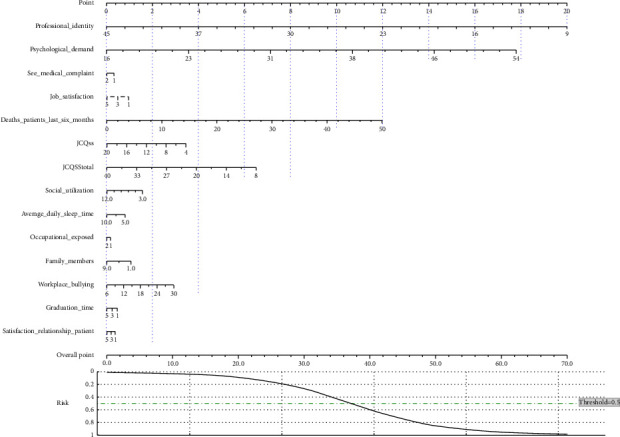
Logistic regression-based nomogram. Top 14 predictors with the largest coefficient absolute values are shown.

This study further demonstrates an LR-based nomogram using the Python method proposed by H. Hong and Hong [60], as shown in Figure 9. It can be found that the nomogram assigns a score to each influencing factor and then the individual scores are summed up to obtain the total score, thereby interpreting the prediction results. This approach has the advantage of being intuitive and easy to understand. Thus, it is widely used in health-related research. However, the disadvantage of nomogram is that the additive nature of the feature effects assumes that the predictors are independent, making it difficult to deal with complexly coupled sets of input variables. In contrast, applying interpretable machine learning not only identifies critical predictors but also captures the nonlinear relationship between the predictors and workplace violence. By isolating the manipulated variables, ALE reveals how and to what extent these factors influence the risk of workplace violence, which facilitates the implementation of more precise intervention strategies by care managers.

## 5. Discussion

### 5.1. Intelligent Prediction and Analysis of Workplace Violence Risk

The results demonstrate that the LightGBM model achieved superior predictive accuracy for workplace violence risk assessment by comprehensively analyzing three key dimensions: demographic characteristics, nurse-related, and work-related factors. It was found that work-related factors play a significant role in workplace violence, with nurse-specific issues being the primary focus. Furthermore, the analysis indicates a nonlinear relationship with workplace violence, informing the development of targeted intervention strategies.

### 5.2. Influencing Factors of Workplace Violence Among Junior Nurses

#### 5.2.1. Work-Related Factors

First, our analysis found that having seen medical complaints is one of the most important factors that increase the risk of workplace violence, in line with non-Chinese regional research [61]. The result also aligns with the emotional contagion theory [62], indicating that negative emotions can be transmitted among nurses through observing others' behavior. When junior nurses experience negative emotions such as fear and tension due to complaints, other nurses who witness this process may unconsciously experience similar negative emotions due to empathy [63]. It not only reduces the job potential of nurses and limits their job performance but also leads to increased nurses' distrust, avoiding excessive contact and aggravating nurse–patient conflicts [64]. Therefore, it is suggested that managers should pay attention to the psychological wellbeing of nurses who have been complained about and witnesses and provide them with prompt psychological counseling, professional training, and specific guidance, thereby addressing patient concerns more effectively and preventing nurse–patient conflicts.

In addition, this study found a significant positive association between occupational exposure and workplace violence. This phenomenon may be due to the negative emotions, such as fear, anxiety, and self-blame, caused by occupational exposure [65]. When not properly addressed, these negative emotions may cause an intense psychological impact on junior nurses and be devastating to their professional confidence. This may lead to fear and trepidation in caring for patients. Persistent anxiety during clinical practice may increase mistrust between nurses and patients and the risk of adverse nursing events, which may contribute to the development of workplace violence. This finding also aligns with Spanish studies [66]. Hence, nursing managers are supposed to strengthen training for occupational exposure prevention, formulate emergency plans, and establish a tracking mechanism for postoccupational exposure.

Moreover, junior nurses who have worked in the emergency department generally face a higher risk of workplace violence, a finding congruent with previous research [2, 67, 68]. This phenomenon may be intertwined with the emergency department's high-pressure, high-risk environment, intensifying the conflict between nurses and patients or their families [69]. Therefore, hospital managers should take adequate measures to provide violence prevention training, establish an effective conflict resolution mechanism, improve the working environment, and enhance security.

Specifically, the findings reveal a notable nonlinear relationship between Daily_round_trip_time and the risk of workplace violence. The workplace violence risk increases when roundtrip time is below 0.6 h or exceeds 1.2 h, whereas durations between 0.6 and 1.2 h act as a protective factor. However, limited empirical evidence exists regarding the mechanisms through which nurses' commute duration modulates workplace violence risk. A possible explanation is that nurses with short commute times live near hospitals and face the pressure of higher housing prices, leading to an increased risk of conflict with patients because of nurses' perceived insecurity and increased chances of working overtime. Long roundtrip times may cause chronic fatigue and reduced patience in nurses, and social isolation weakens social support networks. Based on these findings, it is recommended that nursing managers should implement diverse intervention strategies, such as offering flexible working and remote collaboration options for junior nurses with long roundtrip times, providing safe accommodation subsidies for short-time groups and designing roundtrip social programs such as group commuting shuttles for moderate groups to enhance social support and reduce workplace violence.

#### 5.2.2. Nurse-Related Factors

This study also found a nonlinear correlation between psychological demand and workplace violence risk prediction. Specifically, the risk is low in the 15–30 range, rises sharply in the 30–40 range, and remains high after 45. The finding aligns with the Conservation of Resources (CORs) theory [70, 71], which posits that emotional exhaustion arises when psychological demands surpass coping resources. This emotional exhaustion can lead junior nurses to more conflicts with patients or their families. A high psychological burden in nursing may lead to decision-making fatigue, weakening nurses' sensitivity and responsiveness to potential violent signals. This may result in their inability to promptly identify and respond to situations that may lead to violence, further exacerbating this vicious circle, increasing the risk of workplace violence, and negatively impacting both mental health and job performance. The findings suggest that nursing managers should use predictive models and applications to dynamically assess nurses' psychological demands and understand their impact on workplace violence risk.

Previous studies have mainly explored the negative impact of the occurrence of workplace violence on professional identity and job satisfaction [72]. Interestingly, this research has uncovered that professional identity and job satisfaction are negatively correlated with workplace violence in a nonlinear manner. High professional identity and job satisfaction among nurses reduced workplace violence, consistent with the findings from studies in northeastern and coastal China [73]. This may result from increased nurses' professional identity and job satisfaction, which eases their perception of stress and reduces negative behaviors caused by stress. Research indicates that nurses with higher professional identity and job satisfaction show greater work motivation and commitment [74]. These factors contribute to active thinking and practice in clinical work, flexible response to work emergencies, and reduced workplace violence risk. Therefore, based on the workplace violence prediction model, nursing managers should consider providing career planning and guidance, creating opportunities for learning and promotion, recognizing and rewarding achievements, and fostering a supportive work environment for nurses with a professional identity score below 33 points and job satisfaction score below 3 points. These interventions are anticipated to enhance their professional identity and job satisfaction, ultimately reducing the risk of workplace violence.

### 5.3. Implications on Nursing Management

This study has substantially improved over traditional linear analysis methods by developing a dynamic model for predicting workplace violence. The model effectively captures the nonlinear relationships among complex factors and sensitively identifies crucial risk factors. In addition, the model provides a userfriendly and easy-to-promote application, available in Supporting Information 2, that supports nurses dynamically and real-time monitoring of workplace violence risk. It also helps managers more accurately understand the specific needs of each nurse and design and implement targeted interventions to prevent workplace violence effectively. The code related to the study is accessible at https://github.com/llanjun/WPV-Risk-Analysis.

### 5.4. Limitations

Nevertheless, this study still has certain limitations. First, this study relies on cross-sectional data and does not use time series or longitudinal panel data. Second, the considered predictors did not cover patient-side information, especially the patient's history of violence, personality profile, disease progression, and other aspects highlighted in pivotal review studies [8, 30]. Third, the study sample included only junior nurses from 90 tertiary-level hospitals in Sichuan Province, which may not accurately represent the workplace violence experienced by nurses in hospitals across other regions, thereby limiting the applicability of the results. Ultimately, because this study employed a supervised learning approach, it is subject to time constraints. This means algorithms and methodologies must be regularly updated to maintain model accuracy and ensure findings remain current.

Therefore, future works will follow up with the junior nurse investigated in this study to overcome both limitations while maintaining multicenter data sources and obtaining longitudinal datasets with multivariate time series. Based on this, further policy, organizational, environmental, and patient-side predictors will be collected. Longitudinal causal discovery and interpretable machine learning techniques, such as the longitudinal Linear Non-Gaussian Acyclic Model (LiNGAM) [75], can also be employed to conduct empirical research and obtain richer managerial insights. Future studies will include nurses from multiple regional and level hospitals to improve the representativeness of the results. To keep the approach contemporary, federated learning [76] and fine-tuning pretrained models [77] are crucial solutions. They enable multiple entities to collaborate on developing standard models without sharing local data. Each subject can also train models using private data, thus gaining knowledge that is not limited to the local area.

## 6. Conclusion

In this study, medical complaints, psychological demands, professional identity, and exposure experience are found to be the most critical predictors of workplace violence experienced by junior nurses. The proposed interpretable machine learning framework identifies several important nonlinear effects, particularly the possible U-shaped effect of daily roundtrip time on workplace violence and the stepwise negative effect of professional identity and job satisfaction. In addition, the research model offers a visual guide for future investigations into modular interventions. It empowers nursing managers to address unexpected workplace violence better, providing them with scientific tools and methods for preventing, controlling, and managing such incidents, especially for junior nurses.

## Figures and Tables

**Table 1:** Related research on workplace violence and the considered influencing factors.

Author (year)	Junior nurses	Methods	Important factors
Jonsdottir et al. (2022) [33]	×	Binomial and Poisson regression	Income, working hours, shifts, etc.
Sun, Zhang, and Cao (2022) [37]	×	Logistic regression	Working years, night shift time, working environment, etc.
Sun et al. (2022) [34]	×	Logistic regression	Degree, low income, working hours, depression, etc.
Qi et al. (2022) [35]	×	Logistic regression	Working years, medical errors, depression, anxiety, etc.
Nøland et al. (2021) [36]	×	Generalized estimating equations	Gender, years of working, department, etc.
Zhang et al. (2022) [38]	×	Structural equation modeling	Abuse supervision perception, anxiety, depression, etc.
Yan et al. (2023) [39]	×	Logistic regression	Hospital grade, professional title, history of hypertension, etc.
Tian et al. (2020) [40]	×	Logistic regression	Shift work, department, working hours, hospital grade, etc.
Al-Maskari, Al‐Busaidi, and Al‐Maskari (2020) [41]	×	Logistic regression	Non-Omani nurses, educational qualifications, etc.
Yang et al. (2021) [42]	×	Logistic regression	Working years, working place, caring for infected patients, etc.

**Table 2:** Chi-square and *t*-test results.

Variables	Not experienced or witnessed violence (*n* = 3351)	Experienced or witnessed violence (*n* = 2312)	*χ* ^2^ */t*	*p*
Gender			**9.657**	**0.002**
Man = 1	224 (6.7%)	206 (8.9%)		
Woman = 2	3127 (93.3%)	2106 (91.1%)		
Hukou			**11.795**	**< 0.001**
Countryside	2480 (74.0%)	1615 (69.9%)		
City	871 (26.0%)	697 (30.1)		
Left_behind_children			**17.742**	**< 0.001**
Yes = 1	1605 (47.9%)	1239 (53.6%)		
No = 2	1746 (52.1%)	1073 (46.4%)		
Religious			**9.753**	**0.002**
Yes = 1	166 (5.0%)	160 (6.9%)		
No = 2	3185 (95.0%)	2152 (93.1%)		
Live_with_parents			**14.754**	**< 0.001**
Yes = 1	1777 (53.0%)	1106 (47.8%)		
No = 2	1574 (47.0%)	1206 (52.2%)		
Graduation_time			**81.967**	**< 0.001**
≤ 5 = 1	223 (6.7%)	163 (7.1%)		
3-4 = 2	462 (13.8%)	386 (16.7%)		
2-3 = 3	876 (26.1%)	738 (31.9%)		
1-2 = 4	1017 (30.3%)	701 (30.3%)		
< 1 = 5	773 (23.1%)	324 (14.0%)		
Level_training_hospital			2.906	0.088
Class3B = 1	505 (15.1%)	311 (13.5%)		
Class3A = 2	2846 (84.9%)	2001 (86.5%)		
Degree			**30.596**	**< 0.001**
Technical secondary school education = 1	78 (2.3%)	64 (2.8%)		
College = 2	2503 (74.7%)	1572 (68.0%)		
Bachelor degree or above = 3	770 (23.0%)	676 (29.2%)		
Occupational_exposed			**205.513**	**< 0.001**
Yes = 1	1416 (42.3%)	1425 (61.6%)		
No = 2	1935 (57.7%)	887 (38.4%)		
Working_hours_per_week			**50.469**	**< 0.001**
Less than 41 = 1	1039 (31.0%)	537 (23.2%)		
41–55 = 2	1893 (56.5%)	1431 (61.9%)		
56–70 = 3	349 (10.4%)	258 (11.2%)		
More than 70 = 4	70 (2.1%)	86 (3.7%)		
Sleep_quality			**152.995**	**< 0.001**
Feel good about yourself = 1	861 (25.7%)	375 (16.2%)		
Self-feeling normal = 2	2076 (62.0%)	1400 (60.6%)		
Feel bad about yourself = 3	414 (12.3%)	537 (23.2%)		
Lunch_break			**13.946**	**< 0.001**
Yes = 1	2363 (70.5%)	1522 (65.8%)		
No = 2	988 (29.5%)	790 (34.2%)		
Alcohol_consumption			**12.182**	**< 0.001**
Yes = 1	55 (1.6%)	70 (3.0%)		
No = 2	3296 (98.4%)	2242 (97.0%)		
Smoke			**14.694**	**< 0.001**
Yes = 1	40 (1.2%)	59 (2.6%)		
No = 2	3311 (98.8%)	2253 (97.4%)		
Exercise			**22.481**	**< 0.001**
Yes = 1	1233 (36.8%)	710 (30.7%)		
No = 2	2118 (63.2%)	1602 (69.3%)		
Chronic_disease			**19.616**	**< 0.001**
Yes = 1	85 (2.5%)	109 (4.7%)		
No = 2	3266 (97.5%)	2203 (95.3%)		
Which_chronic_disease			**24.643**	**< 0.001**
No chronic disease = 0	3267 (97.5%)	2204 (95.3%)		
Hypertension = 1	6 (0.18%)	2 (0.1%)		
Allergic asthma = 2	1 (0.02%)	5 (0.2%)		
Other = 3	77 (2.3%)	101 (4.4%)		
Satisfaction_relationship_patient			**198.197**	**< 0.001**
Very dissatisfied = 1	16 (0.5%)	11 (0.5%)		
Dissatisfied = 2	11 (0.3%)	36 (1.6%)		
Normal = 3	955 (28.5%)	1002 (43.3%)		
Satisfied = 4	1986 (59.3%)	1146 (49.6%)		
Very satisfied = 5	383 (11.4%)	117 (5.0%)		
Experienced_medical_complaint			**77.263**	**< 0.001**
Yes = 1	53 (1.6%)	135 (5.8%)		
No = 2	3298 (98.4%)	2177 (94.2%)		
See_medical_complaint			**573.983**	**< 0.001**
Yes = 1	1151 (34.3%)	1542 (66.7%)		
No = 2	2200 (65.7%)	770 (33.3%)		
Job_satisfaction			**383.117**	**< 0.001**
Very dissatisfied = 1	126 (3.8%)	232 (10.0%)		
Dissatisfied = 2	433 (12.9%)	550 (23.8%)		
Normal = 3	1438 (42.9%)	1084 (46.9%)		
Satisfied = 4	1177 (35.1%)	413 (17.9%)		
Very satisfied = 5	177 (5.3%)	33 (1.4%)		
Future_professional_status			**96.446**	**< 0.001**
Below current levels = 1	158 (4.7%)	240 (10.4%)		
Remain unchanged = 2	884 (26.4%)	720 (31.1%)		
Higher than the current level = 3	2309 (68.9%)	1352 (58.5%)		
Aim_policy			**64.029**	**0.001**
No = 0	1385 (41.3%)	714 (30.9%)		
Yes = 1	1966 (58.7%)	1598 (69.1%)		
Aim_higher_class_hospital			**4.527**	**0.033**
No = 0	998 (29.8%)	750 (32.4%)		
Yes = 1	2353 (70.2%)	1562 (67.6%)		
Aim_big_city			**10.414**	**0.001**
No = 0	1505 (44.9%)	1139 (49.3%)		
Yes = 1	1846 (55.1%)	1173 (50.7%)		
Aim_love_nursing			**99.775**	**< 0.001**
No = 0	2427 (72.4%)	1937 (83.8%)		
Yes = 1	924 (27.6%)	375 (16.2%)		
Aim_parents_will			**4.556**	**0.033**
No = 0	2655 (79.2%)	1885 (81.5%)		
Yes = 1	696 (20.8%)	427 (18.5%)		
Aim_nursing_ability			**30.225**	**< 0.001**
No = 0	956 (28.5%)	819 (35.4%)		
Yes = 1	2395 (71.5%)	1493 (64.6%)		
Aim_other			**8.109**	**0.004**
No = 0	3245 (96.8%)	2205 (95.4%)		
Yes = 1	106 (3.2%)	107 (4.6%)		
Economic_area_1			0.657	0.418
No = 0	1338 (39.9%)	948 (41.0%)		
Yes = 1	2013 (60.1%)	1364 (59.0%)		
Economic_area_2			1.575	0.210
No = 0	2843 (84.8%)	1933 (83.6%)		
Yes = 1	508 (15.2%)	379 (16.4%)		
Economic_area_3			1.303	0.254
No = 0	2790 (83.3%)	1898 (82.1%)		
Yes = 1	561 (16.7%)	414 (17.9%)		
Economic_area_4			**4.950**	**0.026**
No = 0	3100 (92.5%)	2174 (94.0%)		
Yes = 1	251 (7.5%)	138 (6.0%)		
Economic_area_5			0.874	0.350
No = 0	3333 (99.5%)	2295 (99.3%)		
Yes = 1	18 (0.5%)	17 (0.7%)		
Marital_1			1.724	0.189
No = 0	303 (9.0%%)	186 (8.0%)		
Yes = 1	3048 (91.0%)	2126 (92.0%)		
Marital_2			1.058	0.304
No = 0	3066 (91.5%)	2133 (92.3%)		
Yes = 1	285 (8.5%)	179 (7.7%)		
Marital_3			1.710	0.191
No = 0	3333 (99.5%)	2305 (99.7%)		
Yes = 1	18 (0.5%)	7 (0.3%)		
Standardized_train_methods_1			**7.318**	**0.007**
No = 0	3296 (98.4%)	2250 (97.3%)		
Yes = 1	55 (1.6%)	62 (2.7%)		
Standardized_train_methods_2			1.643	0.200
No = 0	1732 (51.7%)	1235 (53.4%)		
Yes = 1	1619 (48.3%)	1077 (46.6%)		
Standardized_train_methods_3			0.261	0.609
No = 0	1674 (50.0%)	1139 (49.3%)		
Yes = 1	1677 (50.0%)	1173 (50.7%)		
Department_1			**49.997**	**< 0.001**
No = 0	2514 (75.0%)	1535 (66.4%)		
Yes = 1	837 (25.0%)	777 (33.6%)		
Department_2			0.392	0.531
No = 0	2319 (69.2%)	1618 (70.0%)		
Yes = 1	1032 (30.8%)	694 (30.0%)		
Department_3			2.917	0.088
No = 0	2985 (89.1%)	2092 (90.5%)		
Yes = 1	366 (10.9%)	220 (9.5%)		
Department_4			2.859	0.091
No = 0	3197 (95.4%)	2227 (96.3%)		
Yes = 1	154 (4.6%)	85 (3.7%)		
Department_5			2.440	0.118
No = 0	2831 (84.5%)	1988 (86.0%)		
Yes = 1	520 (15.5%)	324 (14.00%)		
Department_6			**13.674**	**< 0.001**
No = 0	3066 (91.5%)	2176 (94.1%)		
Yes = 1	285 (8.5%)	136 (5.9%)		
Department_7			**6.777**	**0.009**
No = 0	3194 (95.3%)	2236 (96.7%)		
Yes = 1	157 (4.7%)	76 (3.3%)		
Age	22.94 ± 1.597	23.18 ± 1.585	**−5.526**	**< 0.001**
Monthly_household_income	2989.47 ± 1550.489	3027.16 ± 1535.053	−0.905	0.366
Family_members	3.90 ± 1.242	3.78 ± 1.255	**3.461**	**< 0.001**
Average_monthly_salary_income	2651.17 ± 1008.140	2809.84 ± 1100.966	**−5.605**	**< 0.001**
Average_daily_sleep_time	7.002 ± 0.8256	6.764 ± 0.8256	**10.638**	**< 0.001**
Psychological_demand	32.13 ± 4.272	34.41 ± 5.590	**−17.321**	**< 0.001**
Professional_identity	33.94 ± 5.442	31.02 ± 5.639	**19.562**	**< 0.001**
Decision_latitude	73.78 ± 9.506	69.64 ± 10.408	**15.490**	**< 0.001**
Adaptive_performance	50.36 ± 6.547	48.55 ± 6.604	**10.141**	**< 0.001**
RQ_14_total	50.54 ± 8.142	47.53 ± 8.199	**13.611**	**< 0.001**
Workplace_bullying	12.41 ± 4.546	13.81 ± 4.726	**−11.227**	**< 0.001**
Social_utilization	8.30 ± 1.855	7.78 ± 1.778	**10.509**	**< 0.001**
JCQSStotal	30.46 ± 4.867	28.14 ± 5.193	**17.159**	**< 0.001**
JCQss	14.64 ± 2.804	13.22 ± 3.111	**17.913**	**< 0.001**
JCQcs	15.82 ± 2.461	14.92 ± 2.600	**13.223**	**< 0.001**
JCQsd	39.94 ± 4.767	38.08 ± 4.915	**14.252**	**< 0.001**
JCQda	33.84 ± 6.032	31.56 ± 6.886	**13.183**	**< 0.001**
APF1	18.56 ± 2.972	17.73 ± 3.085	**10.209**	**< 0.001**
APF2	31.79 ± 4.147	30.82 ± 4.226	**8.570**	**< 0.001**
RSfactor10	35.30 ± 5.919	33.15 ± 5.905	**13.401**	**< 0.001**
RSfactor4	15.24 ± 2.591	14.38 ± 2.730	**12.040**	**< 0.001**
Average_patients_day_shift	9.05 ± 5.890	10.16 ± 6.248	**−6.788**	**< 0.001**
Deaths_patients_last_six_months	1.02 ± 2.481	1.44 ± 3.119	**−5.695**	**< 0.001**
Daily_roundtrip_time	0.91 ± 0.539	0.96 ± 0.603	**−2.811**	**< 0.001**
Average_length_day_shift	8.17 ± 0.74	8.29 ± 0.78	**−6.031**	**< 0.001**
Day_shift_weekly_frequency	3.50 ± 1.13	3.42 ± 1.13	**2.499**	**0.012**
Night_shift_weekly_frequency	1.93 ± 0.99	2.05 ± 1.02	**−4.279**	**< 0.001**
Average_length_night_shift	8.31 ± 1.27	8.45 ± 1.27	**−4.112**	**< 0.001**
Nurse_hours_dying_patient	10.12 ± 49.91	12.90 ± 43.17	**−2.241**	**0.025**

*Note:* Statistically significant variables are bolded, and more details can be found in the Supporting Information 1.

**Table 3:** Basic classification measurements.

Truth	Prediction	
	0	1
0	True negative (TN)	False positive (FP)
1	False negative (FN)	True positive (TP)

**Table 4:** Performance of machine learning models for predicting workplace violence risk in junior nurses.

Model	Precision	Recall	*F1*	*AUROC*	*AUPRC*	*Brier*	*Accuracy*
XGB	0.541	0.86	0.665	0.755	0.688	0.224	0.628
GBDT	0.685	0.541	0.605	0.752	0.689	0.199	0.696
RF	0.643	0.634	0.638	0.747	0.678	0.206	0.692
LR	0.572	0.621	0.596	0.701	0.633	0.22	0.638
CatBoost	0.562	0.788	0.656	0.742	0.682	0.241	0.646
LightGBM	0.65	0.689	**0.669**	**0.761**	**0.692**	**0.198**	**0.708**

*Note:* The critical measurements are italic, and optimal performance is shown in bold font.

**Table 5:** Five-fold cross validation results.

Model	Precision	Recall	F1	AUROC	AUPRC	Brier	Accuracy
XGB	0.522 (0.005)	0.865 (0.011)	**0.651 (0.003)**	0.75 (0.005)	0.662 (0.016)	0.228 (0.002)	0.622 (0.007)
GBDT	0.635 (0.018)	0.54 (0.014)	0.583 (0.007)	0.742 (0.008)	0.649 (0.015)	**0.2 (0.003)**	0.685 (0.009)
RF	0.606 (0.007)	0.663 (0.018)	0.633 (0.01)	0.751 (0.01)	0.665 (0.019)	0.204 (0.002)	**0.686 (0.006)**
LR	0.57 (0.017)	0.614 (0.027)	0.591 (0.017)	0.703 (0.015)	0.616 (0.015)	0.218 (0.004)	0.653 (0.014)
CatBoost	0.544 (0.003)	0.806 (0.024)	0.65 (0.008)	0.743 (0.006)	0.654 (0.014)	0.246 (0.004)	0.645 (0.004)
LightGBM	0.603 (0.001)	0.678 (0.014)	0.638 (0.007)	**0.754 (0.006)**	**0.668 (0.011)**	0.201 (0.002)	**0.686 (0.007)**

## Data Availability

Due to the sensitive nature of the questions asked in this study, survey respondents were assured raw data would remain confidential and would not be shared. If necessary, please contact xyanwa@163.com or weixuemei750324@163.com; the authors will provide the trained model pickle files to assist with transfer learning and federated learning development.

## Supporting Information

Supporting Information 1 The Supporting Information file 1 presents the variable details and measurements, as a support to Table 2.

Supporting Information 2 The Supporting Information file 2 contains the workplace violence prediction application.
