# Supplementary material for: Identifying the Most Critical Predictors of Workplace Violence Experienced by Junior Nurses: An Interpretable Machine Learning Perspective
Source: J Nurs Manag. 2025 Apr 2;2025:5578698. doi: 10.1155/jonm/5578698 (PMC11981708; doi:10.1155/jonm/5578698)
Supplement: Supporting Information 1 — The Supporting Information file 1 presents the variable details and measurements, as a support to Table 2. [file 5578698.f1.docx]

# Supplementary Material

Table S1. Variable details and naming principles

| Variable | Variable abbreviation | Meaning | Measurement |
| --- | --- | --- | --- |
| Age | Age | The actual age of the individual. | General Demographic Questionnaire |
| Average month income | Average_monthly_salary_income | Refers to the individual average monthly wage income (CNY). | General Demographic Questionnaire |
| Gender | Gender | Gender, including male or female. | General Demographic Questionnaire |
| Household | Hukou | Refers to the official registration record of the permanent residence of the individual registered with the government. | General Demographic Questionnaire |
| Degree | Degree | Refers to the level of education achieved by individuals in the formal education system, including technical secondary school, junior college, undergraduate degree, or above. | General Demographic Questionnaire |
| Sleep time (h) | Average_daily_sleep_time | The total hours that an individual sleeps each day. | General Demographic Questionnaire |
| Psychological Demand | Psychological_Demand | Refers to the internal needs to maintain mental health and achieve individual growth. | Job Content Questionnaire (JCQ) |
| Professional identity | Professional_identity | Refers to the individual's cognitive and emotional sense of belonging to their occupation, and the consistency with the values and behavioral norms of the profession. | Professional Identity Questionnaire (PIQ) |
| Decision Latitude | Decision_Latitude | Refers to the individual's ability of choices to process information, evaluate alternatives, predict consequences, and make decisions. | Job Content Questionnaire (JCQ) |
| Adaptive Performance | Adaptive_performance | Refers to the ability of an individual or organization to adjust and optimize their behavior to maintain work performance when facing environmental changes, new technologies, job role changes, or uncertainty. | Adaptive Performance Questionnaire (APQ) |
| Resilience question | RQ_14_Total | Individuals' ability to adapt, recover, or grow in the face of adversity, stress, disaster, or other forms of challenge. | Resilience Scale-14 (RS-14) |
| Job satisfaction | Job_satisfaction | Refers to the overall feelings of employees on their working environment, work content, salary, promotion opportunities, relationships between colleagues, and management methods. | General Demographic Questionnaire |
| Patients number | Average_patients_day_shift | The average number of patients an individual nurse is responsible for during the day shift. | General Demographic Questionnaire |
| Deaths patients | Deaths_patients_last_six_months | Number of patient mortality in nursing care in the last six months of work. | General Demographic Questionnaire |
| Commuting time (h) | Daily_roundtrip_time | Daily commute time (hours). | General Demographic Questionnaire |
| Each day shift (h) | Average_length_day_shift | The duration of each day shift (hours). | General Demographic Questionnaire |
| See medical complaints | See_medical_complaint | Whether a junior nurse has seen colleagues being subject to medical complaints. | General Demographic Questionnaire |
| Occupational exposure | Occupational_exposed | Refers to whether an individual has ever experienced occupational exposure (e.g., needle injury or radiation). | General Demographic Questionnaire |
| Satisfaction with patients | Satisfaction_relationship_patient | An individual's satisfaction with their relationship with the patient. | General Demographic Questionnaire |
| Left experience | Left_behind_children | Whether the individual left parents or guardians during childhood. | General Demographic Questionnaire |
| Religious | Religious | Whether the individual has religious beliefs or participates in religious activities. | General Demographic Questionnaire |
| Family size | Family_members | Refers to the number of people in a family: the parents, children, grandparents, etc. | General Demographic Questionnaire |
| Live with parents | Live_with_parents | Refers to whether the individual lives with their parents. | General Demographic Questionnaire |
| Work tenure after graduation (year) | Graduation_time | Refers to the length of time (years) of the individual engaged in nursing work. | General Demographic Questionnaire |
| Average household income | Monthly_household_income | Refers to the average monthly salary income of a family member. | General Demographic Questionnaire |
| Future professional status | Future_professional_status | Refers to the judgment of the future prospects of the major. | General Demographic Questionnaire |
| Sleep Quality | Sleep_quality | It refers to sleep efficiency and recovery function, which involves multiple dimensions, including sleep duration, depth, continuity, and the associated physical and psychological recovery processes. | General Demographic Questionnaire |
| Lunch break | Lunch_break | Lunch break criteria: a short rest or sleep during the day (mostly from noon to early afternoon). | General Demographic Questionnaire |
| Alcohol | Alcohol_consumption | Drinking standard: white wine or red wine up to one or two times or beer up to 200 milliliters, drink at least two times a week, drink continuously for more than six months. | General Demographic Questionnaire |
| Smoke | Smoke | Smoking criteria: at least one cigarette per day for more than six consecutive months or a cumulative smoking of 150 cigarettes or more. | General Demographic Questionnaire |
| Exercise | Exercise | Refers to planned and repetitive physical activities, such as running, yoga, swimming, hiking, etc. | General Demographic Questionnaire |
| Chronic disease | Chronic_disease | Refers to the general term of diseases that do not constitute infection and have long-term accumulation to form disease morphological damage, such as cardiovascular and cerebrovascular diseases, cancer, diabetes, chronic respiratory diseases, etc. | General Demographic Questionnaire |
| Which_chronic_disease | Which_chronic_disease | Refers to the specific identification of chronic diseases experienced by an individual. | General Demographic Questionnaire |
| Hospital-Level | Level_training_hospital | It refers to the classification of hospitals according to the complexity of medical services, the perfection of facilities and equipment, the strength of technical strength, teaching, and scientific research ability. | General Demographic Questionnaire |
| Weekly working hours (h) | Working_Hours_per_Week | Time of work per week (hours). | General Demographic Questionnaire |
| Day shifts per week | Day_shift_Weekly_frequency | The number of day shifts per week. | General Demographic Questionnaire |
| Night shifts per week | Night_shift_Weekly_frequency | The number of night shifts per week. | General Demographic Questionnaire |
| Each night shift(h) | Average_length_night_shift | The length of each night shift (hours). | General Demographic Questionnaire |
| End-of-life care hours (h) | Nurse_hours_dying_patient | The length (hours) of individual care for terminal patients in the last six months. | General Demographic Questionnaire |
| Suffer medical complaint | Experienced_medical_complaint | Refers to whether an individual has suffered a medical complaint. | General Demographic Questionnaire |
| Professional bullying | Workplace_Bullying | It means the psychological or physical harassment, assault, or other forms of improper treatment of another employee by an individual or group through continuous and systematic negative behavior in the workplace. | Workplace violence scale(WVS) |
| Social support | JCQSStota | Refers to the emotional, information, physical, or service help and support that an individual receives from their social network. | Job Content Questionnaire(JCQ) |
| Social support utilization degree | Social_utilization | The available social support resources of an individual. | Social Support Rating Scale(SSRS) |
| Leadership support | JCQss | Leadership support | Job Content Questionnaire(JCQ) |
| Colleagues support | JCQcs | Colleagues support | Job Content Questionnaire(JCQ) |
| Skill discretion | JCQsd | Skill discretion | Job Content Questionnaire(JCQ) |
| Decision authority | JCQda | Decision authority | Job Content Questionnaire(JCQ) |
| Adapting to diverse cultures and complex interrelationship | APF1 | Adapting to diverse cultures and complex interrelationships | Adaptive Performance Questionnaire (APQ) |
| Dealing with work pressure and emergency | APF2 | dealing with work pressure and emergency | Adaptive Performance Questionnaire (APQ) |
| Ability | RSfactor10 | Ability | Resilience Scale-14 (RS-14) |
| Positive perception | RSfactor4 | Positive perception | Resilience Scale-14 (RS-14) |
| Policy requirement | Aim_policy | Is the main reason for choosing standardized training is the rigid requirement of national policy？ | General Demographic Questionnaire |
| Work in a third-class A hospital | Aim_higher_class_hospital | I want to work in a third-class A hospital. | General Demographic Questionnaire |
| Work in big cities | Aim_big_city | I want to work in big cities through standardized training opportunities. | General Demographic Questionnaire |
| Love nursing work | Aim_love_nursing | I love nursing work. | General Demographic Questionnaire |
| Parents' will | Aim_parents_will | It's parents' will. | General Demographic Questionnaire |
| Improve nursing abilities | Aim_nursing_ability | I want to improve my nursing abilities. | General Demographic Questionnaire |
| Other reasons | Aim_other | Other reasons | General Demographic Questionnaire |
| Economic area | Economic_area_1 | Southern Sichuan | General Demographic Questionnaire |
| Economic area | Economic_area_2 | Northeast Sichuan | General Demographic Questionnaire |
| Economic area | Economic_area_3 | Chengdu plain area | General Demographic Questionnaire |
| Economic area | Economic_area_4 | The western area of Panzhihua | General Demographic Questionnaire |
| Economic area | Economic_area_5 | Northwest Sichuan | General Demographic Questionnaire |
| Emergency department rotation | Department_1 | Refers to whether an individual has ever had a rotation in the emergency department. | General Demographic Questionnaire |
| Intensive care unit rotation | Department_2 | Whether an individual has ever been rotated in an intensive care unit. | General Demographic Questionnaire |
| Obstetrics or pediatric rotation | Department_3 | Refers to whether an individual has ever been in obstetrics or pediatric rotation. | General Demographic Questionnaire |
| Operating room rotation | Department_4 | Refers to whether the individual has ever been rotated in the operating room. | General Demographic Questionnaire |
| Surgical rotation | Department_5 | Refers to whether an individual has ever had a surgical rotation. | General Demographic Questionnaire |
| Internal medicine rotation | Department_6 | Refers to whether an individual has ever been rotated in internal medicine. | General Demographic Questionnaire |
| Administration department rotation | Department_7 | Whether an individual has ever rotated in the administration. | General Demographic Questionnaire |
| Unmarried | Marital_1 | Unmarried | General Demographic Questionnaire |
| Married | Marital_2 | Married | General Demographic Questionnaire |
| Else | Marital_3 | Else | General Demographic Questionnaire |
| In-hospital training (in progress) | Standardized_train_methods_1 | in-hospital training (in progress) | General Demographic Questionnaire |
| In-hospital training (contract system) | Standardized_train_methods_2 | In-hospital training (contract system) | General Demographic Questionnaire |
| Social people | Standardized_train_methods_3 | Social people | General Demographic Questionnaire |

Table S2. Descriptive statistics of variables

| **Variables** | **N(%) / M±SD** | **Variables** | **N(%) / M±SD** | **Variables** | **N(%) / M±SD** |
| --- | --- | --- | --- | --- | --- |
| **Demographic** | | **Nurse-related** | | **Work-related** | |
| **Age** | 23.04±1.60 | **Average_daily_sleep_time** | 6.91±0.83 | **Average_patients_day_shift** | 9.50±6.08 |
| **Average_monthly_salary_income** | 2715.95±1049.838 | **Psychological_Demand** | 33.06±4.98 | **Deaths_patients_last_six_months** | 1.19±2.77 |
| **Gender** |  | **Professional_identity** | 32.75±5.71 | **Daily_roundtrip_time** | 0.93±0.57 |
| Man=1 | 430(7.6%) | **Decision_Latitude** | 72.09±10.09 | **Average_length_day_shift** | 8.22±0.76 |
| Woman=2 | 5233(92.4%) | **Adaptive_performance** | 49.62±6.63 | **See_medical_complaint** |  |
| **Hukou** |  | **RQ_14_Total** | 49.31±8.30 | Yes=1 | 2693(47.6%) |
| Countryside=1 | 4095(72.3%) | **Job_satisfaction** |  | No=2 | 2970(52.4%) |
| City=2 | 1568(27.7%) | Very dissatisfied=1 | 358(6.3%) | **Occupational_exposed** |  |
| **Degree** |  | Dissatisfied=2 | 983(17.4%) | Yes=1 | 2841(50.2%) |
| Technical Secondary School Education=1 | 142(2.5%) | Normal=3 | 2522(44.5%) | No=2 | 2822(49.8%) |
| College=2 | 4075(72.0%) | Satisfied=4 | 1590(28.1%) | **Satisfaction_relationship_patient** |  |
| Bachelor degree or above=3 | 1446(25.5%) | Very satisfied=5 | 210(3.7%) | Very dissatisfied=1 | 27(0.5%) |
| **Left_behind_children** |  | **Future_professional_status** |  | Dissatisfied=2 | 47(0.8%) |
| Yes=1 | 2844(50.2%) | Below current levels=1 | 398(7.0%) | Normal=3 | 1957(34.6%) |
| No=2 | 2819(49.8%) | Remain unchanged=2 | 1604(28.3%) | Satisfied=4 | 3132(55.3%) |
| **Religious** |  | Higher than the current level=3 | 3661(64.6%) | Very satisfied=5 | 500(8.8%) |
| Yes=1 | 326(5.8%) | **Sleep**_**quality** |  | **Level_training_hospital** |  |
| No=2 | 5337(94.2%) | Feel good about yourself=1 | 1236(21.8%) | Class3B=1 | 816(14.4%) |
| **Family**_**members** | 3.85±1.25 | Self-feeling normal=2 | 3476(61.4%) | Class3A=2 | 4847(85.6%) |
| **Live_with_parents** |  | Feel bad about yourself=3 | 951(16.8%) | **Working_Hours_per_Week** |  |
| Yes=1 | 2883(50.9%) | **Lunch**_**break** |  | Less than 41=1 | 1576(27.8%) |
| No=2 | 2780(49.1%) | Yes=1 | 3885(68.6%) | 41-55=2 | 3324(58.7%) |
| **Graduation_time** |  | No=2 | 1778(31.4%) | 56-70=3 | 607(10.7%) |
| ≤5=1 | 386(6.8%) | **Alcohol_consumption** |  | More than 70=4 | 156(2.8%) |
| 3-4=2 | 848(15.0%) | Yes=1 | 125(2.2%) | **Day_shift_Weekly_frequency** | 3.47±1.13 |
| 2-3=3 | 1614(28.5%) | No=2 | 5538(97.8%) | **Night_shift_Weekly_frequency** | 1.98±1.00 |
| 1-2=4 | 1718(30.3%) | **Smoke** |  | **Average_length_night_shift** | 8.37±1.27 |
| ＜1=5 | 1097(19.4%) | Yes=1 | 99(1.7%) | **Nurse_hours_dying_patient** | 11.26±47.27 |
| **Average_household_income** | 3004.86±1544.181 | No=2 | 5564(98.3%) | **Experienced_medical_complaint** |  |
| **Economic_area_1** |  | **Exercise** |  | Yes=1 | 188(3.3%) |
| NO=0 | 2286(40.4%) | Yes=1 | 1943(34.3%) | No=2 | 5475(96.7%) |
| YES=1 | 3377(59.6%) | No=2 | 3720(65.7%) | **Department_1** |  |
| **Economic_area_2** |  | **Chronic_disease** |  | NO=0 | 4049(71.5%) |
| NO=0 | 4776(84.3%) | Yes=1 | 194(3.4%) | YES=1 | 1614(28.5%) |
| YES=1 | 887(15.7%) | No=2 | 5469(96.6%) | **Department_2** |  |
| **Economic_area_3** |  | **Which_chronic_disease** |  | NO=0 | 3937(69.5%) |
| NO=0 | 4688(82.8%) | no chronic disease=0 | 5471(96.6%) | YES=1 | 1726(30.5%) |
| YES=1 | 975(17.2%) | hypertension=1 | 8(0.1%) | **Department_3** |  |
| **Economic_area_4** |  | allergic asthma =2 | 6(0.1%) | NO=0 | 5077(89.7%) |
| NO=0 | 5274(93.1%) | other=3 | 178(3.1%) | YES=1 | 586(10.3%) |
| YES=1 | 389(6.9%) | **Workplace_Bullying** | 12.98±4.67 | **Department_4** |  |
| **Economic_area_5** |  | **Social_utilization** | 8.09±1.84 | NO=0 | 5424(95.8%) |
| NO=0 | 5628(99.4%) | **JCQSStotal** | 29.51±5.13 | YES=1 | 239(4.2%) |
| YES=1 | 35(0.6%) | **JCQss** | 14.06±3.02 | **Department_5** |  |
| **Marital_1** |  | **JCQcs** | 14.45±2.557 | NO=0 | 4819(85.1%) |
| NO=0 | 489(8.6%) | **JCQsd** | 39.18±4.91 | YES=1 | 844(14.9%) |
| YES=1 | 5174(91.4%) | **JCQda** | 32.91±6.49 | **Department_6** |  |
| **Marital_2** |  | **APF1** | 18.22±3.05 | NO=0 | 5242(92.6%) |
| NO=0 | 5199(91.8%) | **APF2** | 31.39±4.21 | YES=1 | 421(7.4%) |
| YES=1 | 464(8.2%) | **RSfactor10** | 34.42±6.01 | **Department_7** |  |
| **Marital_3** |  | **RSfactor4** | 14.89±2.68 | NO=0 | 5430(95.9%) |
| NO=0 | 5638(99.6%) | **Aim_policy** |  | YES=1 | 233(4.1%) |
| YES=1 | 25(0.4%) | NO=0 | 2102(37.1%) |  |  |
| **Standardized_train_methods_1** |  | YES=1 | 3561(62.9%) |  |  |
| NO=0 | 5546(97.9%) | **Aim_higher_class_hospital** |  |  |  |
| YES=1 | 117(2.1%) | NO=0 | 1748(30.9%) |  |  |
| **Standardized_train_methods_2** |  | YES=1 | 3915(69.1%) |  |  |
| NO=0 | 2967(52.4%) | **Aim_big_city** |  |  |  |
| YES=1 | 2696(47.6%) | NO=0 | 2644(46.7%) |  |  |
| **Standardized_train_methods_3** |  | YES=1 | 3019(53.3%) |  |  |
| NO=0 | 2813(49.7%) | **Aim_love_nursing** |  |  |  |
| YES=1 | 2850(50.3%) | NO=0 | 4364(77.1%) |  |  |
|  |  | YES=1 | 1299(22.9%) |  |  |
|  |  | **Aim_parents_will** |  |  |  |
|  |  | NO=0 | 4540(80.2%) |  |  |
|  |  | YES=1 | 1123(19.8%) |  |  |
|  |  | **Aim_nursing_ability** |  |  |  |
|  |  | NO=0 | 1775(31.3%) |  |  |
|  |  | YES=1 | 3888(68.7%) |  |  |
|  |  | **Aim_other** |  |  |  |
|  |  | NO=0 | 5450(96.2%) |  |  |
|  |  | YES=1 | 213(3.8%) |  |  |
