# Supplementary material for: Identifying the Most Critical Predictors of Workplace Violence Experienced by Junior Nurses: An Interpretable Machine Learning Perspective
Source: J Nurs Manag. 2025 Apr 2;2025:5578698. doi: 10.1155/jonm/5578698 (PMC11981708; doi:10.1155/jonm/5578698)
Supplement: Supporting Information 2 — The Supporting Information file 2 contains the workplace violence prediction application. [file 5578698.f2.docx]

# Supplementary Material 2


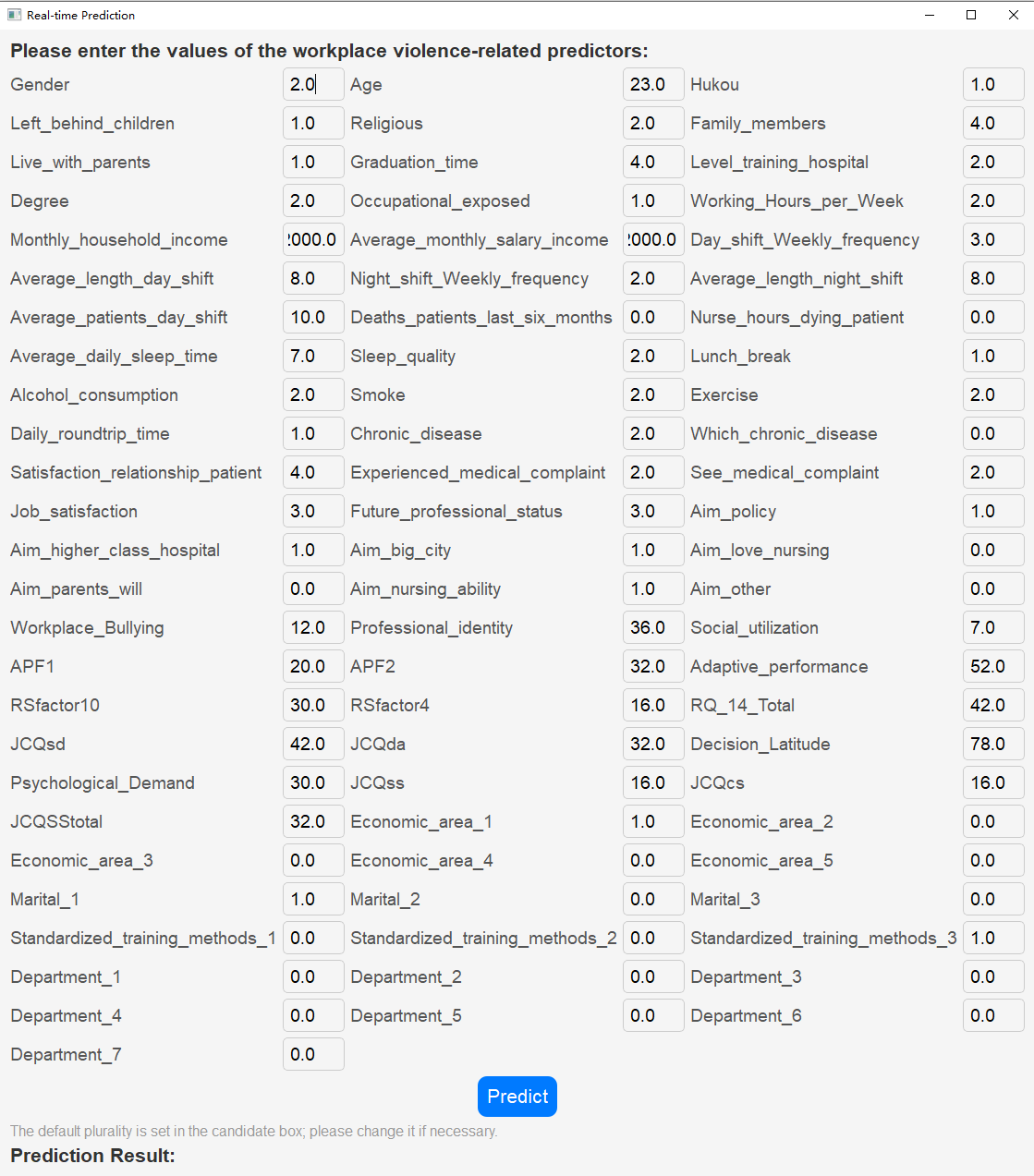


Figure S1. Workplace Violence Prediction Application

We have uploaded this application to https://github.com/llanjun/WPV-Risk-Analysis.
